# A Cursory Inquiry into Some of the Principal Causes of Mortality among Children, with a View to Assist in Ameliorating the State of the Rising Generation, in Health, Morals, and Happiness: To Which Is Added, an Account of the Universal Dispensary for Sick Indigent Children

**Published:** 1817-03

**Authors:** 


*A Cursory Inquiry into some of the principal Causes of Mortality among Children, with a View to assist in ameliorating the State of the rising Generation, in Health, Morals, and Happiness: to which is added, an Account of the Universal Dispensary for sick indigent Children.* By JOHN BUNNELL DAVIS, M. D. Licentiate of the Royal College of Physicians, Senior Physician to the London Dispensary, &c. pp. 72. London, 1817.

THIS little work, although not strictly medical, is intimately connected with medical science, inasmuch as it professes to advocate the establishment of a charitable institution for the treatment of children only, when labouring under illness, which may in its progress facilitate the investigation of infantile diseases, enable us to ascertain more accurately the principal causes of them, and conduct us to a knowledge of more successful means for their prevention and cure than we have yet been able to attain by the present promiscuous admission of adults and children in our medical charities. However questionable may be the advantages of a minute subdivision of the healing art, when the views of a practitioner are exclusively directed, and his education solely confined to his own particular branch of practice, no one can doubt the utility that may arise from rescuing the children of the poor from the ignorant and illiterate empiric, to whom they are in the present state of things too often consigned, and bringing them under the management of an enlightened and judicious physician. It must be confessed, that in the lower ranks of life, where the operation of various moral and physical causes, prejudicial to health and destructive to life, are more extensively active, the effects of these upon the tender frame and feeble constitution of infants, have been too much disregarded, whilst in sickness the most preposterous methods are resorted to for a cure, which ignorant superstition can devise and blind credulity listen to. Much of this evil arises, no doubt, from a vulgar prejudice, not confined, we are sorry to say, to the lowest classes, that physicians know nothing of children's diseases, and that an old nurse is the best doctor. Were no other good effected by this new establishment, than the removal of this prejudice, we should say much had been done; but we feel convinced that great actual benefit will be conferred on medical science, from the opportunity thus afforded, of forming general principles by cautious induction from the numerous facts which must present themselves in so extensive a collection of cases, as will by this means be brought together and concentrated in one spot. By the active zeal and unvaried exertions of the author of this pamphlet, by interesting some of the most illustrious characters in the objects of his views, by producing, through the means of a spirited and animated address, an universal conviction of the benefit to be derived from his intended plan, he has at length succeeded in establishing a Dispensary for Poor Children.

The "Cursory Inquiry," although well adapted for conveying to the general reader, correct notions of the causes unfavourable to the successful rearing up of the progeny of the poor, and an intelligible account of the best means of obviating them, contains nothing that is new or unknown to the medical world. Every physician's experience must have made him acquainted with the former, and have afforded him frequent opportunities of practising the latter. Whatever possible benefit is to be derived to medical science, or improvement made in the healing art, by means of this exclusive establishment, we may fairly anticipate, when we see the avidity with which the poor in this metropolis embrace the advantages held out to them by this charity, and when we consider the zeal and ability of the medical officers to whom the management of the concern is entrusted. Within seven months during which the Dispensary has been open, 1021 children have been admitted, of whom 19 appear to have died.

"Of the children that died, six were under 1 year of age, of whom two had colliquative diarrhœa, from mesenteric disease; four influenza, and one fits: eight under two years, of whom two had colliquative diarhœa, from mesenteric disease; one the measles, one dropsy on the brain, one general dropsy, and two hooping-cough: four under 4 years, of whom one had the measles, one a diseased spleen, one water on the head, one a pulmonary affection after influenza, and one hooping cough, after croup; one under 6 years, who had dropsy, combined with dysentery, after the scarlet fever; and one under 8 years of age, who had water on the head.

"And it is worth remarking, that of the fatal epidemical influenza of this season, only four children have died out of 215 admitted into the Dispensary, which is a strong and evident confirmation of the benefit resulting from open admission when the case is urgent, and immediate assistance required.

"It appears from the above list, that the most prevalent diseases among children during the winter, have been the peripneumony, or influenza of infants, measles, hooping cough, scarlet fever, mumps, and a variety of acute glandular affections. In November, the influenza began to attack children in different districts of the metropolis, and the adjacent villages. It first produced high inflammation in the lining of the trachea and its numerous ramifications, then in the lungs; and in the fatal instances of the disease, a change of structure ensued in the membrane, together with effusion into the bronchial tubes. Influenza was frequently observed to precede the measles, and sometimes the croup; and in the milder cases of it, an obstinate cough and periodical fever, which resisted the ordinary means of treatment, frequently followed. Whenever it preceded the appearance of the measles, this disease was invariably severe, and unless the inflammatory diathesis occasioned by influenza was overcome prior to the eruption, a loss of sensorial power, with symptoms denoting approaching effusion, ensued.

"Notwithstanding the number of severe cases of measles admitted into the Dispensary, only one ended fatally; but in all those preceded by influenza, it was necessary to resort to the most vigorous treatment in the beginning, to avert a fatal termination.

"Is it not probable, that the great mortality from the measles this season in the counties of Sussex and Suffolk, has had its source in an inflammatory diathesis, established by a previous attack of influenza, and which was not removed before the action arising from the introduction of measles supervened?

"A peripneumonic affection, similar to an advanced case of influenza, has latterly constituted a primary disease in hooping-cough, and rendered it unusually intractable. This was the case in the fatal instances recorded of this disorder.

"By the long continuance of a mild temperature of the air, influenza is less formidable than it was at the approach of the cold season. Still, however, it is a predominant disease among children.

"The Reporter never recollects to have seen the mumps and glandular affections of the throat so general as they have been throughout the whole course of the winter.

"Of the cases recorded of scarlet fever, three were followed by the dropsy, and one by dysentery combined with dropsy, which ended unfavourably."

A list is given of women married since 1800, who have attended with their children at the Dispensary, taken promiscuously, in order to show the number of deaths, and the proportion of survivors under twelve years of age in each family. From this, it appears, that to 75 women 415 children were born alive, of whom there died 178; survived to 12 years of age 237: proportion of deaths to births, as 178 to 415.

"Might not those who died of the small-pox, and a large proportion of those that were put to wet-nurse, and brought up by hand, have been saved?

"Of the 178 children that died, 31 were put out to wet-nurse, and 15 brought up by the hand. The small-pox destroyed 21.

"The greater part of the deaths, in the proportion of 3 to 4, happened under one year of age."

